# Real‐World Survival Over 25 Years in Extensive‐Stage Small‐Cell Lung Cancer: The Impact of Immune Checkpoint Inhibitors

**DOI:** 10.1111/1759-7714.70316

**Published:** 2026-06-02

**Authors:** Ryo Ariyasu, Teruhisa Nemoto, Hirokazu Iso, Masahiro Ito, Kyujiro Nibuya, Syunsuke Fujishima, Mayu Sugai, Ryosuke Tsugitomi, Yoshiaki Amino, Ken Uchibori, Noriko Yanagitani, Makoto Nishio

**Affiliations:** ^1^ Department of Thoracic Medical Oncology The Cancer Institute Hospital of Japanese Foundation for Cancer Research Tokyo Japan

**Keywords:** atezolizumab, durvalumab, extensive‐stage small‐cell lung cancer, immune checkpoint inhibitors, overall survival

## Abstract

**Introduction:**

Therapeutic progress for extensive‐stage small‐cell lung cancer (ES‐SCLC) has been limited. Although immune checkpoint inhibitors (ICIs) have been introduced, their real‐world impact on survival remains uncertain. We evaluated 25‐year survival trends and the association between ICI exposure and overall survival (OS).

**Methods:**

Consecutive ES‐SCLC patients treated at a Japanese cancer center between April 1999 and March 2023 were retrospectively reviewed and grouped by diagnosis period: A (1999–2003), B (2004–2008), C (2009–2013), D (2014–2018), and E (2019–2023). OS from diagnosis to death was measured. Kaplan–Meier/log‐rank tests and multivariable Cox models were used. Subgroup analyses included patients aged ≥ 75 years, with PS ≥ 2, and with brain metastases.

**Results:**

A total of 452 patients were included. Median OS in period A was 9.8 months and in periods B–E, it was 12.5, 13.4, 11.8, and 13.6 months; 2‐year OS rates were 6.2%, 18.2%, 18.6%, 17.3%, and 28.9%, respectively. In multivariable analysis, PS ≥ 2 (HR 1.51), receipt of first‐line therapy (HR 0.09), and ICI exposure (HR 0.73; *p* = 0.013) were independently associated with OS. ICI exposure was associated with longer OS (15.2 vs. 12.3 months; 2‐year OS 32.1% vs. 15.9%; *p* < 0.005) among systemically treated patients. In subgroup analyses, ICI‐associated HRs were 0.67 in patients aged ≥ 75 years, 0.69 in PS ≥ 2, and 0.63 in brain metastases.

**Conclusions:**

Real‐world survival in ES‐SCLC improved over 25 years. ICI exposure was independently associated with improved OS, as well as in clinically high‐risk subgroups.

## Graphical abstract

This 25‐year real‐world cohort of Japanese patients with extensive‐stage small‐cell lung cancer showed improved survival over time, with immune checkpoint inhibitor exposure independently associated with longer overall survival, including a consistent benefit direction in clinically high‐risk subgroups.

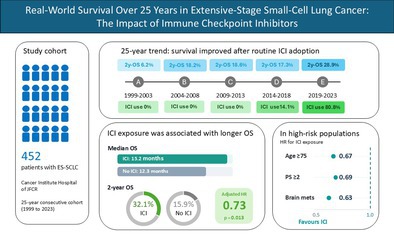

**Abbreviations**
BSC: best supportive care
HR: hazard ratio
ICI: immune checkpoint inhibitors
NSCLC: non‐small cell lung cancer
OS: overall survival
RWD: real‐world data

## 1 Introduction

The treatment landscape for lung cancer, particularly non‐small cell lung cancer (NSCLC), has substantially evolved in recent years. Survival outcomes for selected patients with NSCLC have significantly improved after the advent of molecularly targeted therapies and immune checkpoint inhibitors (ICIs) [1, 2]. In contrast, extensive‐stage small‐cell lung cancer (ES‐SCLC) continues to have limited therapeutic progress, and prognosis remains poor. SCLC is generally considered highly sensitive to cytotoxic chemotherapy [3], and OS gains have been modest despite introduction of regimens including topotecan and amrubicin for relapsed disease [4, 5, 6]. Unlike NSCLC, SCLC rarely harbors actionable oncogenic driver alterations, and the lack of effective molecularly targeted therapies has been a major impediment to achieving durable disease control and improving long‐term outcomes [7].

In the absence of effective molecularly targeted therapies, immunotherapy has been explored as a promising strategy for ES‐SCLC. Small‐cell lung cancer is characterized by a high tumor mutational burden and has, therefore, been considered a potentially immunogenic disease that may respond to ICI [8]. In addition, chemotherapy may enhance the antitumor effects of ICIs by increasing tumor antigen release and promoting a more immunogenic tumor microenvironment through treatment‐induced tumor cell death [9]. In recent years, ICIs have been included in the treatment regimen for ES‐SCLC. In 2019, the anti‐PD‐L1 antibody atezolizumab was approved as first‐line treatment for ES‐SCLC in combination with platinum–etoposide chemotherapy. In 2020, another anti‐PD‐L1 antibody, durvalumab was also approved for first‐line use in combination with the chemotherapy. Both anti‐PD‐L1–based regimens demonstrated an overall survival (OS) benefit compared with chemotherapy alone in pivotal phase III trials [10, 11, 12, 13]. However, the extent to which these benefits translate to routine clinical practice remains to be clarified.

Outcomes observed in clinical trials might not be generally applicable to real‐world populations that include older patients, patients with several comorbidities, or patients with poor performance status. Therefore, validation using real‐world data (RWD) is very important [14]. Prior RWD studies in ES‐SCLC have not consistently demonstrated clear survival improvements [15, 16, 17]. This could be attributed to the fact that several cohorts have been treated prior to the introduction of ICIs. Consequently, the real‐world impact of ICIs on survival in ES‐SCLC has not been fully elucidated, particularly in analyses spanning extended time periods that capture secular changes in treatment patterns.

Therefore, this study aimed to evaluate long‐term survival trends over the past 25 years in Japanese patients diagnosed with ES‐SCLC. Specifically, the association between the introduction of ICIs and survival outcomes across a broad range of clinical backgrounds in a real‐world setting was assessed.

## 2 Material and Methods

### 2.1 Study Design and Patient Population

This retrospective cohort study aimed to evaluate long‐term survival trends among patients with extensive‐stage small‐cell lung cancer (ES‐SCLC) and to assess whether there was an association between exposure to immune checkpoint inhibitors (ICIs) and OS. We used our institutional clinical database to identify consecutive patients diagnosed with ES‐SCLC at the Department of Thoracic Oncology, Cancer Institute Hospital of the Japanese Foundation for Cancer Research. ES‐SCLC was defined as stage IV disease in accordance with the seventh edition of the TNM classification for lung cancer. Eligible patients were those diagnosed between April 1999 and March 2023, along with patients who received systemic anticancer treatment and those who managed with best supportive care (BSC) alone. The data extracted included the age of the patient at diagnosis, sex, smoking history, Eastern Cooperative Oncology Group performance status (ECOG PS), presence of brain metastases, date of diagnosis, date of death, and treatment history. Exposure to ICIs (nivolumab, pembrolizumab, atezolizumab, and durvalumab) was ascertained from the treatment records, and ICI exposure at any line of therapy was included in the analysis. Secular trends were examined by categorizing the patients into five calendar periods based on the year of diagnosis: period A (1999–2003), period B (2004–2008), period C (2009–2013), period D (2014–2018), and period E (2019–2023). OS was defined as the time from the date of diagnosis to death from any cause. Surviving patients at the data cutoff date were censored at the last confirmed follow‐up date (July 2025). Patients lost to follow‐up were censored at the date of last contact. The institutional review board of our hospital approved this study (IRB No. 2025‐GB‐068). This study was conducted following the Declaration of Helsinki established in 2013. The requirement for informed consent was waived because of the retrospective design of the study, and an opt‐out mechanism was provided to the patients.

### 2.2 Statistical Analysis

Kaplan–Meier method was used for estimating OS, and the log‐rank test was used for comparing the survival curves. Moreover, the evaluation of the association between patient characteristics and OS in multivariate analysis was done by performing Cox proportional hazards regression analysis. Statistical significance was defined as a two‐sided *p* < 0.05. All statistical analyses were performed using SPSS Statistics for Windows version 24 (IBM Corp., NY, USA).

## 3 Results

### 3.1 Patient Characteristics

Of the 970 patients diagnosed with SCLC, 452 patients with ES‐SCLC were included in this study. A STROBE‐compliant flow diagram is shown in Figure S1. Among a total of 452 patients with ES‐SCLC treated between 1999 and 2023, 48 were treated in period A, 71 in period B, 101 in period C, 128 in period D, and 104 in period E. Patient characteristics have been summarized in Table 1. The median age gradually increased over time and was highest in period E (70 years); Period E also had the highest proportion of patients aged ≥ 75 years (30.8%). The proportion of female patients also increased over time and was higher in periods D and E (25.8% and 27.9%, respectively). Never‐smokers accounted for a small proportion across all periods (2.3%–6.7%). Poor performance status (ECOG PS ≥ 2) was more commonly observed in periods A and D (35.4% and 29.7%), and period B had the least number of brain metastases at diagnosis (8.5%). Period E had the highest proportion of patients managed with BSC alone (10.6%), whereas recipients of second‐line therapy were lowest in period A (37.5%). Exposure to ICIs was observed from period D onward (14.1% in period D and 80.8% in period E). The period was significantly associated with ICI exposure (chi‐square test, *p* < 0.001). The proportion of patients receiving palliative radiotherapy was lowest in period A (39.6%) but was similar across the other periods (53.9%–60.6%).

**TABLE 1:** Baseline characteristics by period (Groups A–E).

Group		A	B	C	D	E
*N*		48	71	101	128	104
Age (years)	Median	66	67	67	69	70
Age (%)	< 75	38 (79.2)	56 (78.9)	76 (75.2)	100 (78.1)	72 (69.2)
	≥ 75	10 (20.8)	15 (21.1)	25 (24.8)	28 (21.9)	32 (30.8)
Sex (%)	Female	5 (10.4)	12 (16.9)	14 (13.9)	33 (25.8)	29 (27.9)
	Male	43 (89.6)	59 (83.1)	87 (86.1)	95 (74.2)	75 (72.1)
Smoking	Never	2 (4.2)	4 (5.6)	4 (4.0)	3 (2.3)	7 (6.7)
	Formerly or unknown	46 (95.8)	67 (94.4)	97 (96.0)	125 (97.7)	97 (93.3)
PS	0–1	31 (64.6)	59 (83.1)	81 (80.2)	90 (70.3)	82 (78.8)
	2–4	17 (35.4)	12 (16.9)	20 (19.8)	38 (29.7)	22 (21.2)
Brain mets (%)	Yes	8 (16.7)	6 (8.5)	21 (20.8)	30 (23.4)	21 (20.2)
	No	40 (83.3)	65 (91.5)	80 (79.2)	98 (76.6)	83 (79.8)
1st line (%)	Yes	47 (97.9)	71 (100.0)	100 (99.0)	125 (97.7)	93 (89.4)
	No	1 (2.1)	0 (0.0)	1 (1.0)	3 (2.3)	11 (10.6)
2nd line (%)	Yes	18 (37.5)	56 (78.9)	77 (76.2)	83 (64.8)	64 (61.5)
	No	30 (62.5)	15 (21.1)	24 (23.8)	45 (35.2)	40 (38.5)
ICI (%)	Yes	0 (0.0)	0 (0.0)	0 (0.0)	18 (14.1)	84 (80.8)
	No	48 (100)	71 (100)	101 (100)	110 (85.9)	20 (19.2)
RT (%)	Yes	19 (39.6)	43 (60.6)	59 (58.4)	69 (53.9)	58 (55.8)
	No	29 (60.4)	28 (39.4)	42 (41.6)	59 (46.1)	46 (44.2)

Abbreviations: ICI, immune checkpoint inhibitor; Mets, metastasis; PS, performance status; RT, radiation therapy.

### 3.2 The Analysis of Overall Survival

**FIGURE 1:**
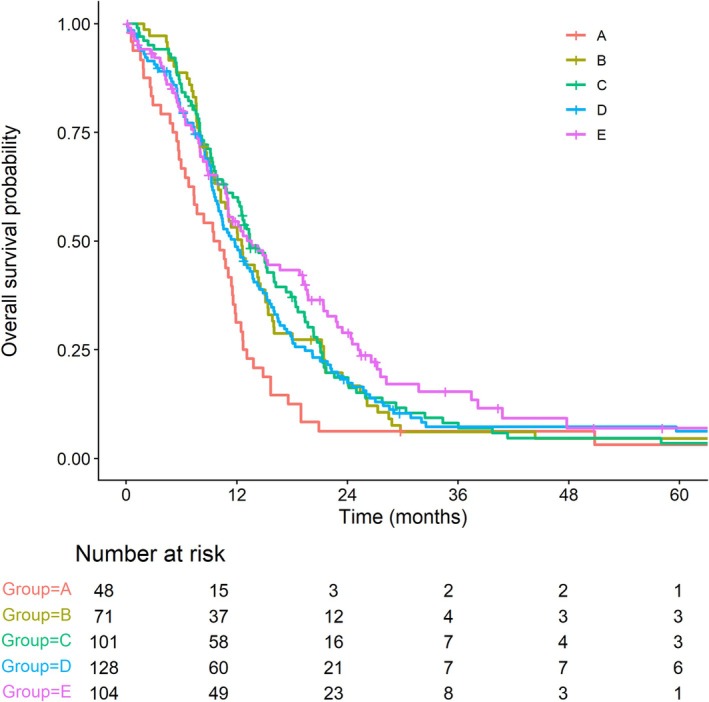
Period wise Kaplan–Meier curves for OS.

The median follow‐up was 11.6 months. Of the 452 patients, 398 experienced an event. Period wise, median OS was 9.8 months in period A, and 12.5, 13.4, 11.8, and 13.6 months in periods B through E, respectively. The corresponding 2‐year OS rates were 6.2% in period A, 18.2% in period B, 18.6% in period C, 17.3% in period D, and 28.9% in period E (Figure 1).

Multivariate Cox regression analyses adjusted for age, sex, smoking status, performance status, brain metastases, and treatment history demonstrated that ECOG PS ≥ 2, receipt of first‐line chemotherapy, and ICI exposure were independently associated with OS. The hazard ratios (HRs) were 1.51 (95% CI, 1.34–1.70) for ECOG PS ≥ 2, 0.09 (95% CI, 0.05–0.17) for receipt of first‐line treatment, and 0.73 (95% CI, 0.57–0.94) for ICI exposure (Table 2).

**TABLE 2:** Multivariable Cox model and subgroup hazard ratios.

Characteristics	Variables	HR	95% CI	*p*
Age	≥ 75 vs. < 75 years	1.16	0.91–1.46	0.232
Sex	Male vs. Female	1.26	0.98–1.61	0.075
Smoking	Former or unknown vs. Never	0.83	0.51–1.33	0.438
PS	2–4 or unknown vs. 0–1	1.51	1.34–1.70	< 0.005
Brain metastasis	Yes vs. No	0.98	0.75–1.27	0.866
1st line treatment	Yes vs. No	0.09	0.05–0.17	< 0.005
ICI treatment	Yes vs. No	0.73	0.57–0.94	0.013

Abbreviations: ICI, immune checkpoint inhibitor; PS, performance status.

**FIGURE 2:**
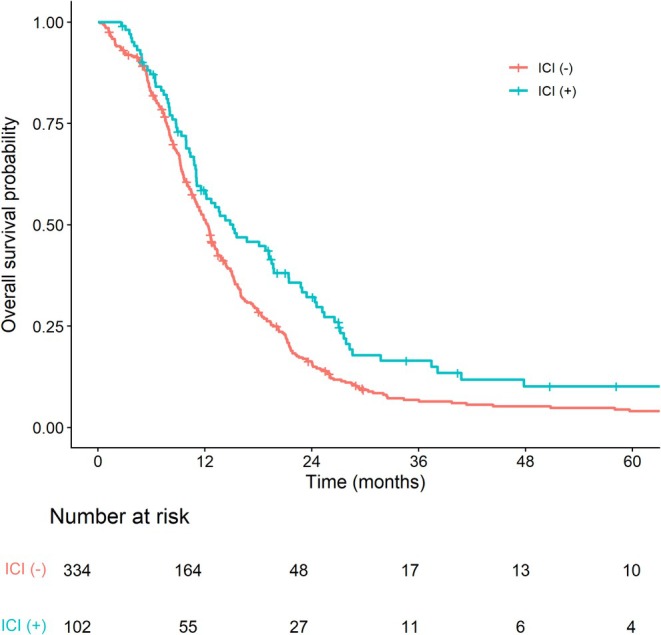
OS by ICI exposure (excluding BSC‐only patients).

OS was compared according to ICI exposure in patients who received systemic treatment (excluding those who were managed with BSC alone). Patients treated with ICIs had significantly longer median OS compared with those who had not been treated with ICIs (15.2 vs. 12.3 months; *p* < 0.005). The corresponding 2‐year OS rates were 32.1% and 15.9%, respectively (Figure 2). The results were consistent when overall survival was calculated from treatment initiation (14.6 vs. 11.6 months; *p* < 0.005). In a time‐dependent Cox proportional hazards model treating ICI exposure as a time‐varying covariate, ICI exposure remained significantly associated with improved overall survival (HR, 0.74; 95% CI, 0.58–0.95; *p* = 0.016).

### 3.3 Subgroup Analysis by Baseline Characteristics

**FIGURE 3:**
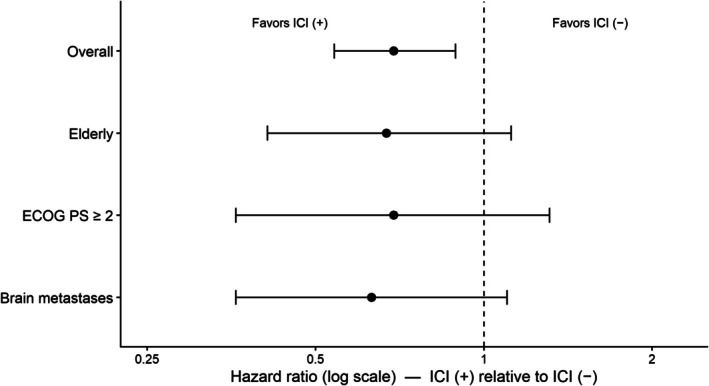
Subgroup analysis of overall survival by ICI exposure (forest plot).

Subgroup analyses were performed in older patients (≥ 75 years; *n* = 103), patients with ECOG PS ≥ 2 (*n* = 96), and those with brain metastases (*n* = 82) to further evaluate the association between ICI exposure and survival among patients with poor prognostic features. ICI exposure was associated with a reduced hazard of death in the overall cohort (HR, 0.69; 95% CI, 0.54–0.89). In subgroup analyses, the HRs for ICI exposure were 0.67 (95% CI, 0.41–1.12) in older patients, 0.69 (95% CI, 0.36–1.31) in patients with ECOG PS ≥ 2, and 0.63 (95% CI, 0.36–1.10) in patients with brain metastases (Figure 3).

**FIGURE 4:**
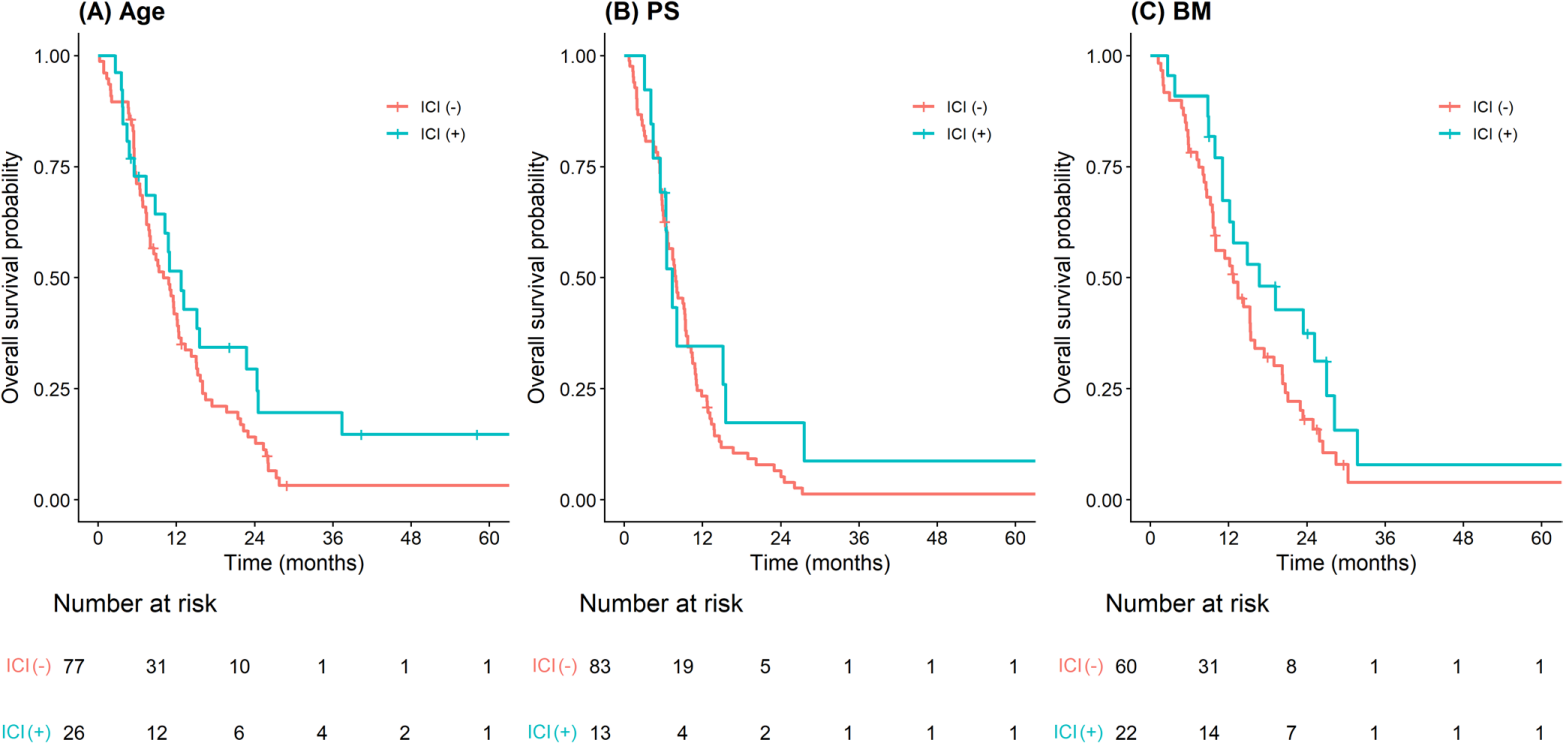
Subgroup Kaplan–Meier curves (older patients, PS ≥ 2, brain metastases).

Median OS (ICI vs. no ICI) was 12.7 versus 10.0 months among older patients, 7.4 versus 7.9 months among patients with ECOG PS ≥ 2, and 16.7 versus 12.7 months among patients with brain metastases. The corresponding 2‐year OS rates (ICI vs. no ICI) were 29.4% versus 14.0%, 17.3% versus 5.2%, and 37.4% versus 18.1%, respectively. Kaplan–Meier curves for these subgroups have been depicted in Figure 4.

## 4 Discussion

In this retrospective cohort study spanning 25 years, long‐term survival trends in Japanese patients with extensive‐stage small‐cell lung cancer (ES‐SCLC) were assessed and the association between ICI exposure and OS was examined. OS was observed to gradually increase over time, with more pronounced increases from 2004 onward and again after 2019. These temporal changes coincided with evolving treatment patterns, including increased use and diversification of second‐line chemotherapy. In addition, the adoption of anti–PD‐L1–based chemoimmunotherapy in routine practice represented a major therapeutic milestone for ES‐SCLC. In Japan, atezolizumab plus platinum–etoposide was approved for ES‐SCLC in 2019, followed by durvalumab in 2020 [10, 12]. In our cohort, there was a substantial increase in ICI exposure in the most recent period, and OS for patients who received ICIs had longer OS compared to those who did not receive ICIs. Furthermore, ICI exposure remained independently associated with improved OS in multivariable analyses when adjusting for key prognostic variables. Collectively, these findings suggest that the inclusion of ICIs in real‐world practice has contributed to improved outcomes in ES‐SCLC.

Though several studies have examined survival trends in SCLC, most real‐world analyses did not capture the full impact of chemoimmunotherapy, since they were conducted predominantly in the pre‐ICI era [15, 16, 17]. A recent nationwide real‐world study from France reported improved survival outcomes following the introduction of immunotherapy, despite incomplete (approximately 55%) ICI uptake in that cohort [18]. The unique contribution of our study lies in its evaluation of a 25‐year consecutive real‐world cohort spanning both the pre‐ICI and post‐ICI eras, with high ICI uptake in the most recent period (80.8%). Among patients who received systemic therapy, 90.3% were treated with ICIs, likely facilitated by Japan's universal health insurance system. This setting may allow a clearer assessment of immunotherapy‐associated outcomes in routine clinical practice. In addition, this study represents one of the first long‐term real‐world evaluations from Asia, where such evidence remains limited, and provides an opportunity to re‐examine the effectiveness of ICIs in ES‐SCLC in the context of regional and potential ethnic diversity.

An important gap between pivotal trials and clinical practice is that patients with poor general condition are frequently excluded due to strict eligibility criteria [19]. Because of the rapidly progressive nature of the disease [3], ES‐SCLC often presents with advanced age, poor performance status, and/or brain metastases in routine care. Therefore, RWD analyses including diverse patients who are more likely to be excluded from clinical trials are particularly important in ES‐SCLC [20]. Such higher‐risk features were common in our cohort, and we observed that the direction of benefit associated with ICIs was preserved across these clinically relevant subgroups. Since analyses incorporating detailed patient backgrounds are scarce, our findings support the broader use of ICIs across the ES‐SCLC population, including high‐risk patients. Nonetheless, confidence intervals in subgroup analyses were wide, and these findings should be interpreted cautiously due to limited power.

The particularly poor outcomes in the earliest period are also noteworthy. These poor outcomes could be attributed to the fact that in period A, uptake of second‐line therapy was lower than in later periods since the key second‐line agents such as topotecan (approved in 2000) and amrubicin (approved in 2002) had only recently become available in Japan [21, 22], and their clinical use was likely still limited. Given that the efficacy of agents such as topotecan or amrubicin for relapsed SCLC has been demonstrated in clinical trials [4, 5, 6], our findings reaffirm the significance of chemotherapy in SCLC management, even in the era of ICIs.

This study has several limitations. First, despite multivariable adjustment, the single‐center retrospective design introduces potential selection bias and residual confounding. In particular, because our institution is a specialized cancer center, the study population may have been enriched for patients who were fit enough to be referred to our hospital, and the quality and consistency of supportive care may also have been higher than in other settings. These factors may have contributed to more favorable survival outcomes and may limit the generalizability of our findings. Second, in this study, overall survival was calculated from the date of diagnosis rather than from the start of first‐line therapy. Although the results were generally consistent when overall survival was calculated from treatment initiation, this approach may still introduce bias because patients with rapidly progressive disease may not have been able to receive adequate treatment. Third, a subset of patients did not receive ICIs even after their approval, which may reflect clinical contraindications, rapid clinical deterioration, or other unmeasured factors that could confound associations with survival. Fourth, the impact of evolving diagnostic practices could not be evaluated. Not only systemic therapy but also diagnostic modalities changed over the years; indeed, the observed rate of brain metastases differed among periods. Fifth, in our cohort, ICI exposure increased substantially in the most recent period, and calendar period was significantly associated with ICI exposure, as expected given the timing of ICI introduction. Because these variables were closely related, some degree of collinearity is possible, and their independent effects should therefore be interpreted cautiously.

## 5 Conclusions

Survival outcomes for patients with ES‐SCLC improved over time in our real‐world cohort. Our results indicate an association between exposure to ICIs and improved OS in the real‐world clinical settings. These findings support consideration of ICIs in routine clinical practice for patients with ES‐SCLC, including clinically high‐risk populations. Future prospective studies are warranted to further evaluate the effectiveness of ICIs in these high‐risk patient groups.

## Author Contributions

**Teruhisa Nemoto:** writing – review and editing. **Syunsuke Fujishima:** writing – review and editing. **Ryo Ariyasu:** conceptualization, methodology, validation, formal analysis, investigation, data curation, writing – original draft, visualization. **Kyujiro Nibuya:** writing – review and editing. **Mayu Sugai:** writing – review and editing. **Hirokazu Iso:** writing – review and editing. **Ryosuke Tsugitomi:** writing – review and editing. **Makoto Nishio:** writing – review and editing, supervision. **Yoshiaki Amino:** writing – review and editing. **Ken Uchibori:** writing – review and editing. **Masahiro Ito:** writing – review and editing. **Noriko Yanagitani:** writing – review and editing.

## Funding

The authors have nothing to report.

## Conflicts of Interest

Ryo Ariyasu received honoraria from Astra Zeneca, Chugai Pharmaceutical, MSD, ONO Pharmaceutical, and Bristol Myers Squib. Ken Uchibori received Grants or contracts from Amgen, Astra Zeneca, PharmaMar, Genmab, Bristol Myers Squib, TAIHO and AnHeart Therapeutics, received honoraria from Astra Zeneca, Amgen, Chugai, Takeda Pharmaceutical, Ono Pharmaceutical, Eli‐Lilly, Bristol Myers Squib, Merck, Daiichi‐Sankyo, MSD, Novartis and Themofisher. Noriko Yanagitani received honoraria from Chugai Pharmaceutical, ONO Pharmaceutical, Bristol‐Myers Squibb, AstraZeneca, Eli Lilly, Pfizer and Daiichi Sankyo Company, Limited. Makoto Nishio received honoraria from ONO Pharmaceutical, Chugai Pharmaceutical, TAIHO Pharmaceutical, Bristol‐Myers Squibb, Daiichi‐Sankyo, Eli Lilly, Merck Sharp & Dohme, Abbvie, Takeda Pharmaceutical, Pfizer, Boehringer Ingelheim, Novartis, Nippon Kayaku, Merck, Janssen and AstraZeneca. The other authors had no competing interests to declare.

## Supporting information


**Figure S1:** A STROBE‐compliant flow diagram.

## Data Availability

The data that support the findings of this study are available on request from the corresponding author. The data are not publicly available due to privacy or ethical restrictions.
