# Supplementary figures and images for: Real‐World Survival Over 25 Years in Extensive‐Stage Small‐Cell Lung Cancer: The Impact of Immune Checkpoint Inhibitors
Source: Thorac Cancer. 2026 Jun 2;17(11):e70316. doi: 10.1111/1759-7714.70316 (PMC13239237; doi:10.1111/1759-7714.70316)

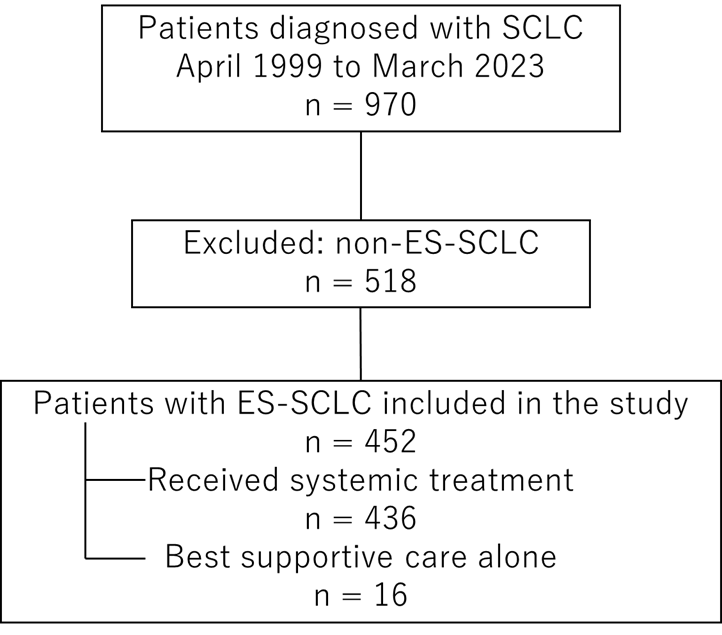

Supplement: Supplementary file 1 — Figure S1: A STROBE‐compliant flow diagram. [file TCA-17-e70316-s001.tif]
